# The growing footprint of artificial intelligence in periodontology & implant dentistry

**DOI:** 10.34172/japid.2023.012

**Published:** 2023-06-11

**Authors:** Adileh Shirmohammadi, Sina Ghertasi Oskouei

**Affiliations:** ^1^Department of Periodontics, Faculty of Dentistry, Tabriz University of Medical Sciences, Tabriz, Iran; ^2^Section of Digital Dentistry, Facutly of Dentistry, Tabriz University of Medical Sciences, Tabriz, Iran

**Keywords:** Artificial intelligence, Dentists, Periodontics, Privacy, Software

 Artificial intelligence (AI) is the simulation of human intelligence processes by machines, especially computer systems. AI has been making waves in various fields, and dentistry is no exception. In recent years, AI has been increasingly used in periodontology and implant dentistry to improve patient outcomes and streamline dental procedures.

 AI has gained recent public prominence with the release of deep-learning models that can generate anything from art to term papers with minimal human intervention. This development has reinvigorated the discussion of AI’s existing and potential roles in all aspects of life. Among the wide range of fields with possible applications of AI, however, medicine and dentistry stand out due to tremendous potentials and equally substantial challenges. AI development has proved successful in solving problems in specific areas by learning distinct thinking mechanisms and perceptions, with a rapidly increasing number of manuscripts that consider some aspect of AI application in medicine and dentistry.^1,2^

 AI is ever-increasing in medicine and dentistry as an assistive tool, becoming a central tenet in providing safe and effective healthcare. More recently, deep learning has been the mainstay of this endeavor, mainly through its applications stemming from the use of artificial neural networks that exhibit a very high degree of complexity,^3^ where large numbers of artificial neurons (or nodes) are connected into layers, and several hundreds or thousands of layers are assembled into specific structures called architectures. Deep learning networks can assess large volumes of data to perform specific tasks, among which electronic health records, imaging data, wearable-device sensor collections, and deoxyribonucleic acid (DNA) sequencing play a prominent role. These are classically used in medical fields for computer-aided diagnoses, personalized treatments, genomic analyses, and treatment response assessments.

 Periodonticsis an important field of dentistry that focuses on the health of periodontium, the tissues that support teeth. Periodontitis is the sixth most prevalent disease worldwide.^4^ AI can help in the early detection of periodontal disease by analyzing radiographs and identifying changes in bone density and periodontal tissue, which allows for earlier intervention and better treatment outcomes. There has been a marked increase in the number of studies published in this field over the last decade. One example is algorithms for diagnosing and predicting the teeth that are compromised with periodontal health. Scott et al^5^ reviewed the literature describing the effect AI has on the diagnosis and epidemiology of periodontitis. Extensive search was performed in April 2022, including studies where AI was employed as an independent variable in the assessment, diagnosis, or treatment of patients with periodontitis. Furthermore, other research efforts showed that AI, with its varied methods and applications, such as machine learning, will change the face of periodontology in the upcoming years.^5-8^

 Implantology is the growing field of dentistry dedicated to dental implants. Implant dentistry involves the placement of artificial tooth roots to support replacement teeth. AI applications are growing in dental implant procedures. One application of AI in implant dentistry is its use in digital three-dimensional (3D) treatment planning for aligning intraoral 3D images with cone-beam computed tomography (CBCT) data in software for surgical evaluation and planning. The current software technology enables accurate 3D planning as per practitioners’ choice. AI can assist in implant planning by analyzing intraoral scan and CBCT data of a patient to determine the optimal location for implant placement, reducing the risk of complications during surgery and improving the success rate of implants. AI can also aid in designing custom-made dental prostheses, such as crowns, bridges, and full-arch restorations. As CBCT is being applied to implant dentistry, the ever-increasing use of this technology produces a critical number of images that can be used for training AI. Revilla-León et al^9^ have reviewed the performance of AI models in implant dentistry for implant type recognition, implant success prediction by using patient risk factors and ontology criteria, and implant design optimization combining finite element analysis calculations and AI models.

 Future directions in implant dentistry could combine CBCT scans with radiographic image data to aid in data analysis and increase the accuracy of implant type recognition. Implementing a special class of deep learning methods, such as 1-shot learning and less-than-1-shot learning, that require fewer data points than neural network models might facilitate implementing and improving AI models for implant dentistry applications.^10^

 In addition to improving diagnosis and treatment planning, AI can assist in patient communication. Chatbots powered by AI technology can answer common questions about dental procedures, reducing wait times for patients seeking information from their dentist.^2^

 However, some challenges still need to be addressed before AI becomes widely adopted in periodontology and implant dentistry. One major concern is data privacy since AI requires access to sensitive patient information such as medical records and imaging data. Additionally, there is a need for more research on how AI algorithms can be trained on diverse datasets to ensure unbiased decision-making.

 Despite these challenges, it is clear that AI has enormous potential to transform the field of periodontology and implant dentistry. As technology advances, we can expect more innovative applications of AI in dental care that will improve patient outcomes while reducing costs and increasing efficiency.

 There is no doubt the body of literature on the applications of AI in dentistry in general, and in periodontology and implant dentistry in particular, will continue to grow, and the* Journal of Advanced Periodontology & Implant Dentistry* (JAPID) welcomes submissions to be published in this area. Although this adds the burden of soliciting peer-reviewers outside the realm of dentistry, including individuals such as data scientists and AI researchers, it is a challenge that the editorial board of JAPID welcomes to advance this exciting interdisciplinary field of health and information technology.

## Competing Interests

 Adileh Shirmohammadi is the Editor-in-Chief of JAPID, and Sina Ghertasi Oskouei serves as an Associate Editor for JAPID. The authors declare that they have no other competing interests with regard to authorship and/or publication of this work.

## References

[1] Haug CJ, Drazen JM (2023). Artificial intelligence and machine learning in clinical medicine, 2023. N Engl J Med.

[2] Lee P, Bubeck S, Petro J (2023). Benefits, limits, and risks of GPT-4 as an AI chatbot for medicine. N Engl J Med.

[3] Goodfellow I, Bengio Y, Courville A. Deep Learning. Cambridge, MA: MIT Press; 2016.

[4] Kassebaum NJ, Bernabé E, Dahiya M, Bhandari B, Murray CJ, Marcenes W (2014). Global burden of severe periodontitis in 1990-2010: a systematic review and meta-regression. J Dent Res.

[5] Scott J, Biancardi AM, Jones O, Andrew D (2023). Artificial intelligence in periodontology: a scoping review. Dent J (Basel).

[6] Kim EH, Kim S, Kim HJ, Jeong HO, Lee J, Jang J (2020). Prediction of chronic periodontitis severity using machine learning models based on salivary bacterial copy number. Front Cell Infect Microbiol.

[7] Huang W, Wu J, Mao Y, Zhu S, Huang GF, Petritis B (2020). Developing a periodontal disease antibody array for the prediction of severe periodontal disease using machine learning classifiers. J Periodontol.

[8] Lee JH, Kim DH, Jeong SN, Choi SH (2018). Diagnosis and prediction of periodontally compromised teeth using a deep learning-based convolutional neural network algorithm. J Periodontal Implant Sci.

[9] Revilla-León M, Gómez-Polo M, Vyas S, Barmak BA, Galluci GO, Att W (2023). Artificial intelligence applications in implant dentistry: a systematic review. J Prosthet Dent.

[10] Yauney G, Rana A, Wong LC, Javia P, Muftu A, Shah P (2019). Automated process incorporating machine learning segmentation and correlation of oral diseases with systemic health. Annu Int Conf IEEE Eng Med Biol Soc.

